# Mixed adenoneuroendocrine carcinoma of the non-ampullary duodenum with mismatch repair deficiency: a rare case report

**DOI:** 10.1007/s00795-022-00324-x

**Published:** 2022-05-20

**Authors:** Yumi Nozawa, Kazuyuki Ishida, Niki Maiko, Atsuko Takada-Owada, Masato Onozaki, Mina Takaoka, Kinichi Matsuyama, Yuhki Sakuraoka, Yoshimasa Nakazato, Keiichi Kubota

**Affiliations:** 1 Department of Diagnostic Pathology, Dokkyo Medical University, 880 Kitakobayashi, Mibu, Tochigi 321-0293 Japan; 2 Second Department of Surgery, Dokkyo Medical University, Mibu, Tochigi Japan; 3 Department of Pathology, Dokkyo Medical University Hospital, Mibu, Tochigi Japan

**Keywords:** Non-ampullary duodenum, Mixed adenoneuroendocrine carcinoma, Neuroendocrine carcinoma, Mismatch repair deficiency, Unique morphology, Immunohistochemistry, Tumorigenesis mechanism

## Abstract

A non-ampullary duodenal mixed adenoneuroendocrine carcinoma (MANEC), consisting of a conventional adenocarcinoma and a neuroendocrine carcinoma (NEC), is exceedingly rare. Moreover, mismatch repair (MMR) deficient tumors have recently attracted attention. The patient, a 75-year-old woman with epigastric pain and nausea, was found to have a type 2 tumor of the duodenum, which was diagnosed on biopsy as a poorly differentiated carcinoma. A pancreaticoduodenectomy specimen showed a well-defined 50 × 48 mm tumor in the duodenal bulb, which was morphologically composed of glandular, sheet-like, and pleomorphic components. The glandular component was a tubular adenocarcinoma, showing a MUC5AC-positive gastric type. The sheet-like component consisted of homogenous tumor cells, with chromogranin A and synaptophysin diffusely positive, and a Ki-67 index of 72.8%. The pleomorphic component was diverse and prominent atypical tumor cells proliferated, focally positive for chromogranin A, diffusely positive for synaptophysin, and the Ki-67 index was 67.1%. The sheet-like and pleomorphic components were considered NEC, showing aberrant expression of p53, retinoblastoma, and p16. Notably, all three components were deficient in MLH1 and PMS2. We diagnosed a non-ampullary duodenal MANEC with MMR deficiency. This tumor has a unique morphology and immunohistochemical profile, and is valuable for clarifying the tumorigenesis mechanism of a non-ampullary duodenal MANEC.

## Introduction

The coexistence of neuroendocrine and non-neuroendocrine components in the same neoplasm, with each component accounting for at least 30% of the neoplasm, is defined as a mixed neuroendocrine non-neuroendocrine neoplasm (MiNEN) [1]. A MiNEN has the highest incidence of mixed adenoneuroendocrine carcinomas (MANEC) combined with adenocarcinomas and neuroendocrine carcinomas [1]. In the gastrointestinal tract, neuroendocrine carcinomas (NEC) and MANECs are more common in the large intestine, followed by the stomach, and less common in the small intestine [2]. In the small intestine, NECs and MANECs are mostly found in the duodenum, but most are ampulla, and non-ampullary duodenal MANECs or NECs are exceedingly rare [3].

A MANEC of the colon has a poorer prognosis than a conventional colonic adenocarcinoma [4, 5]. The molecular analyses of the components of conventional adenocarcinomas and NECs in MANECs of the gastrointestinal tract suggest a common monoclonal origin [6–8]. The clinicopathologic characteristics and molecular features of NECs compared with neuroendocrine tumors have also been clarified [9–11]. Additionally, the tumorigenesis of primary duodenal adenocarcinomas has been actively investigated, and the difference between the clinicopathologic features of gastric and intestinal types has been attracting attention [12–14]. Nevertheless, the characteristics of adenocarcinomas and NECs comprising non-papillary duodenal MANECs, and their tumorigenesis mechanisms, remain to be clarified.

Tumors with a microsatellite instability (MSI) status defined as mismatch repair (MMR) deficiency have been shown to be sensitive to an immune checkpoint blockade with antibodies to programmed death receptor-1 [15]. The immunohistochemical staining of MMR proteins has been shown to provide substantially equivalent information and a more convenient and efficient alternative method for detecting MSI phenotype in an intestinal tract carcinoma [16–18]. MMR-deficient carcinomas are more frequent in the endometrium, stomach, small intestine, and large intestine [19, 20]. Small intestinal carcinomas are rare, although MMR-deficient carcinomas were present in 10–20% of small intestinal carcinomas [21, 22]. It has been suggested that the clinicopathological characteristics and prognosis of small intestinal adenocarcinomas with MMR deficiency differ from those without MMR deficiency [22]. However, the relationship between MMR deficiency and neuroendocrine tumors in the gastrointestinal tract is not well understood. Here, we report the very rare case of a non-ampullary duodenal MANEC with MMR deficiency.

## Case presentation

### Clinical history

A 75-year-old woman developed epigastric pain and nausea over a period of three months. Esophagogastroduodenoscopy revealed an ulcerated tumor in the duodenal bulb and a biopsy specimen showed a poorly differentiated carcinoma. The patient’s tumor marker serum levels, such as those for carcinoembryonic antigen (CEA), carbohydrate antigen 19-9 (CA19-9), pancreatic monoclonal antigen type 2 (Dupan-2) and s-pancreas-1 antigen (Span-1) were within the normal range. Computed tomography suggested lymph node metastasis along the common hepatic artery. No distant metastasis was revealed by positron emission tomography. A pancreaticoduodenectomy was subsequently performed resulting in a clinical diagnosis of duodenal cancer.

Pancreaticoduodenectomy specimens were obtained that were originally prepared from 10% buffered formalin-fixed, paraffin-embedded tissue according to our routine hospital procedure. A histopathological examination was performed using hematoxylin and eosin (HE) staining. Immunohistochemistry was conducted using an autoimmunostainer (Leica BOND-III system: Leica Biosystems, Newcastle, UK). The antibodies we employed are listed in Table 1. Immunohistochemistry was performed on MMR proteins using MLH1, PMS2, MSH2, and MSH6. Negative protein expression (i.e., immunohistochemistry aberrant expression) was defined as the complete absence of nuclear staining within tumor cells in the presence of nuclear staining in internal non-neoplastic cells [18, 23].

**Table 1:** Antigens used for immunohistochemical study

Antigen	Clone	Dilution	Source
Cytokeratin 7	OV-TL 12/30	1:100	Agilent Technologies, Santa Clara, CA
Cytokeratin 20	Ks20.8	1:40	Agilent Technologies, Santa Clara, CA
MUC2	Ccp58	1:100	Leica Biosystems, Nussloch, DE
MUC5AC	CLH2	1:100	Leica Biosystems, Nussloch, DE
MUC6	CLH5	1:100	Leica Biosystems, Nussloch, DE
CDX2	CDX2-88	Ready to use	Abcam, Cambridge, UK
CD10	56C6	Ready to use	Leica Biosystems, Nussloch, DE
Chromogranin A		Ready to use	NICHIREI BIOSCIENCES INC., Tokyo, JP
Synaptophysin	27G12	Ready to use	Leica Biosystems, Nussloch, DE
CD56	CD564	Ready to use	Leica Biosystems, Nussloch, DE
INSM1	A-8	1:200	Santa Cruz Biotechnology, Dallas, TX
SSTR2	EP149	1:50	NICHIREI BIOSCIENCES INC., Tokyo, JP
p53	DO-7	Ready to use	Leica Biosystems, Nussloch, DE
p16	E6H4	Ready to use	Roche Diagnostics K.K., Basel, DE
Retinoblastoma	G3-245	1:50	BD Biosciences, Franklin Lakes, NJ
Ki-67	MIB-1	1:100	Agilent Technologies, Santa Clara, CA
MLH1	ES05	Ready to use	Agilent Technologies, Santa Clara, CA
PMS2	EP51	Ready to use	Agilent Technologies, Santa Clara, CA
MSH2	FE11	Ready to use	Agilent Technologies, Santa Clara, CA
MSH6	EP49	Ready to use	Agilent Technologies, Santa Clara, CA

*INSM1* insulinoma-associated protein 1, *SSTR2* somatostatin receptor subtype 2

### Pathologic findings

**Fig. 1:**
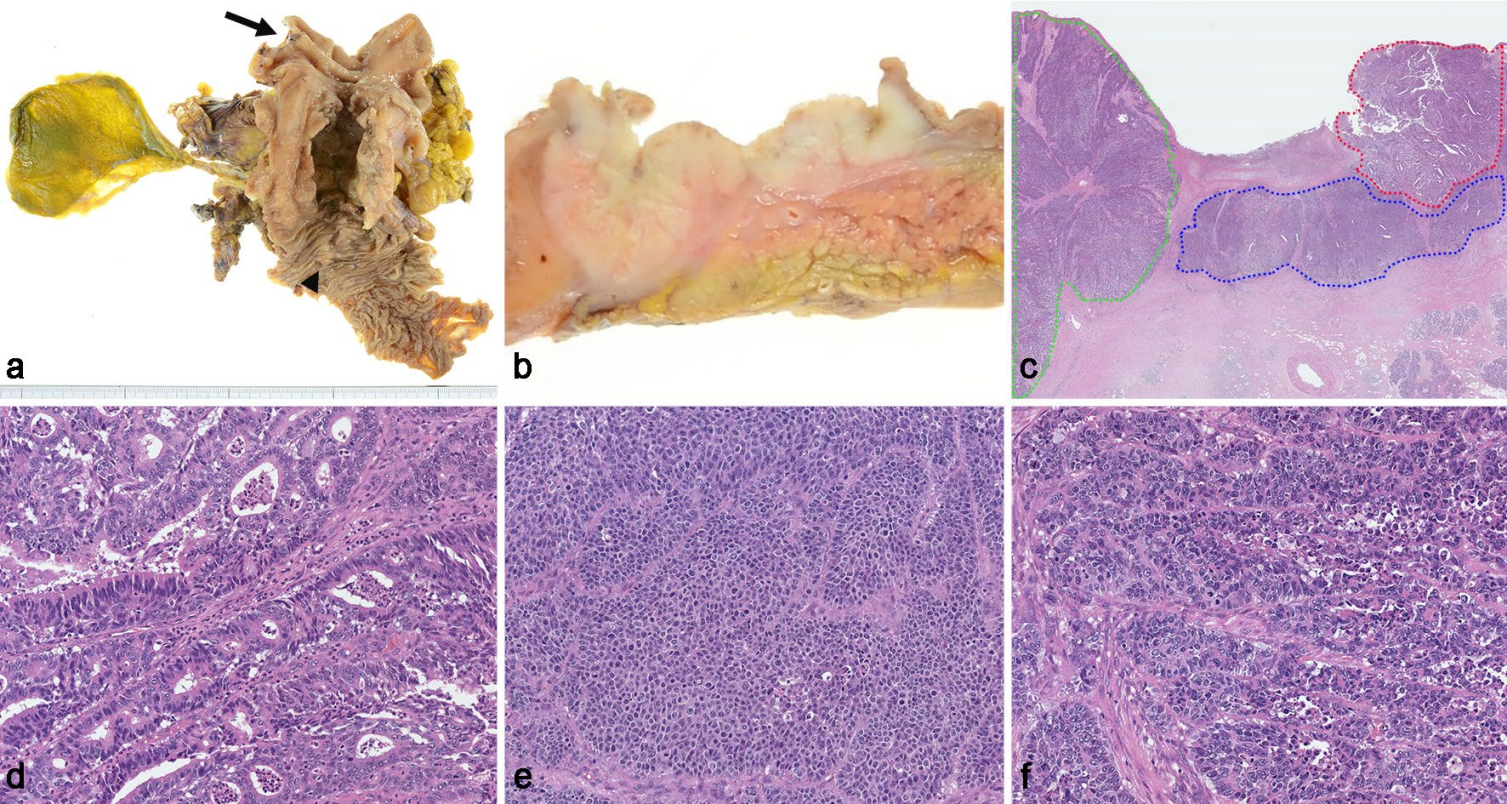
Pathologic findings for mixed adenoneuroendocrine carcinoma. **a** Pancreaticoduodenectomy specimen showed an ulcerative and localized tumor of 50 × 48 mm in the duodenal bulb. The tumor was far from the pylorus (arrow) and ampulla (arrowhead). **b** The cut surface of the resected specimen revealed a solid-milky and well-circumscribed mass. The tumor had not invaded the pancreas. **c** The tumor exhibited three distinct morphological components: glandular component (red), sheet-like component (blue), and pleomorphic component (green). **d** The glandular component was mainly a moderately differentiated tubular adenocarcinoma. **e** The sheet-like component showed medullary growth with a few fibrous stromata. **f** The pleomorphic component formed irregular shaped nests with necrosis. **c**–**f** Hematoxylin and eosin-stained sections. Original magnification: **c** scanning view; **d**–**f** × 200

There was a type 2 tumor in the duodenal bulb that measured 50 × 48 mm. The tumor was separated from the ampulla (Fig. 1a). The cut surface of the tumor showed a milky-white mass with well-defined borders (Fig. 1b). The tumor invaded beyond the muscularis propria without pancreatic invasion. The tumor was histologically observed to have glandular, sheet-like, and pleomorphic components (Fig. 1c). The glandular component, which was clearly distinguishable from the other components and accounted for 30% of the tumor, was a tubular adenocarcinoma with back-to-back glands and cribriform formations, consisting of columnar tumor cells with prominent nucleoli (Fig. 1d). The sheet-like component consisted of tumor cells showing round nuclei, prominent nucleoli, and a high nuclear/cytoplasmic ratio. The cell mitosis was 31 per 2 mm^2^. This component was morphologically suspected to be neuroendocrine differentiation (Fig. 1e). The pleomorphic component showed various structures composed of tumor cells with prominent cytological atypia such as loss of nuclear polarity, nuclear polymorphism, and prominent nucleoli. Small foci of necrosis were detected and the cell mitosis was 40 per 2 mm^2^ (Fig. 1f).

**Fig. 2:**
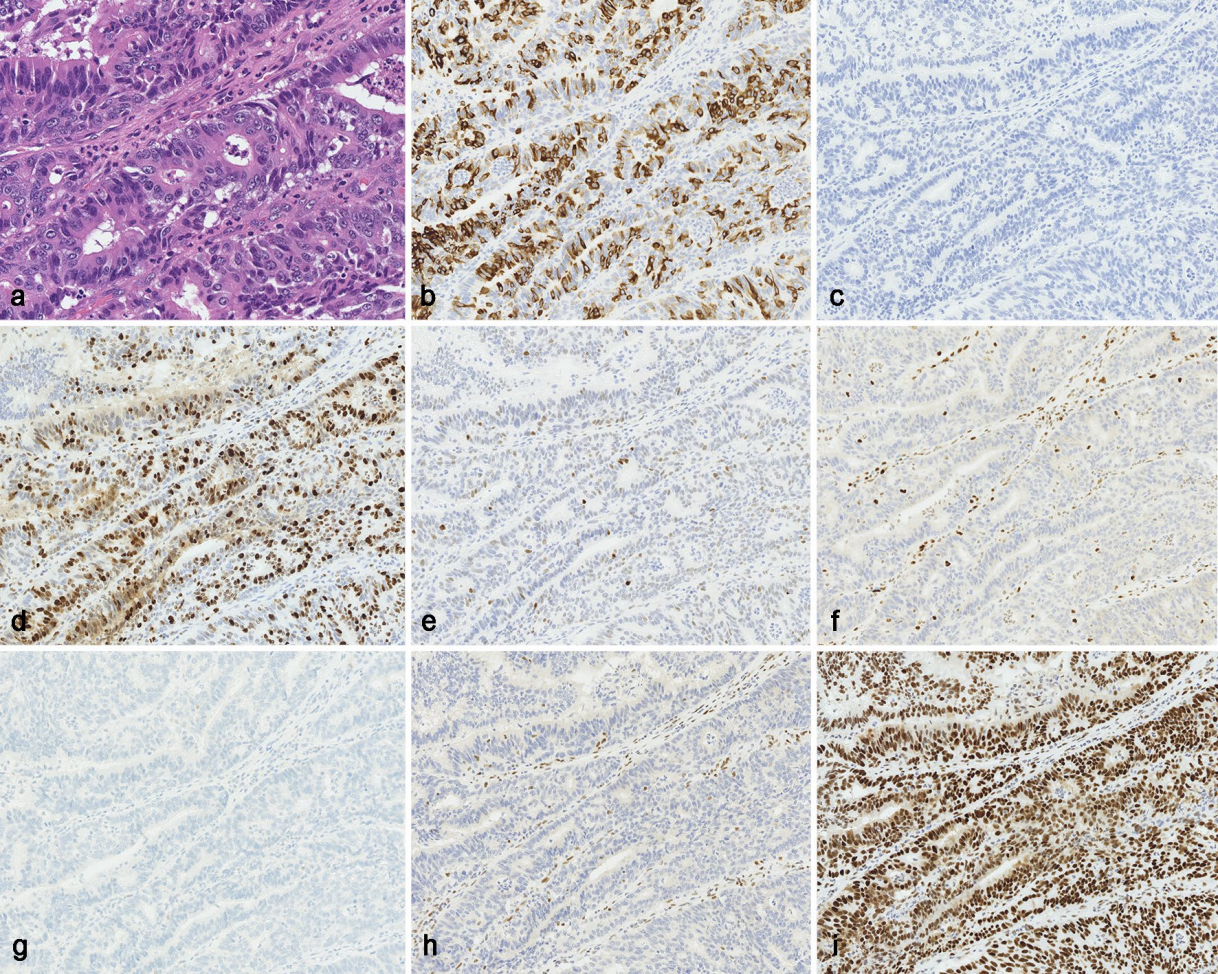
Histological and immunohistochemical findings for the glandular component. **a** The glandular component was composed of columnar cells with cytological atypia. **b** The tumor cells were immunohistochemically positive for MUC5AC. **c** Synaptophysin was negative. **d** The Ki-67 labeling index was 27.7%. **e** p53 had a wild type status showing a low or weak nuclear expression in the tumor cells. **f** Retinoblastoma revealed aberrant expression with complete deletion in tumor cells, while non-tumor cells were not deleted. **g** p16 was negative. Mismatch repair proteins were completely deleted for PMS2 (**h**) and diffusely positive for MLH6 (**i**) in the tumor cells. Original magnification: **a** × 400; **b**–**i** × 200

**Fig. 3:**
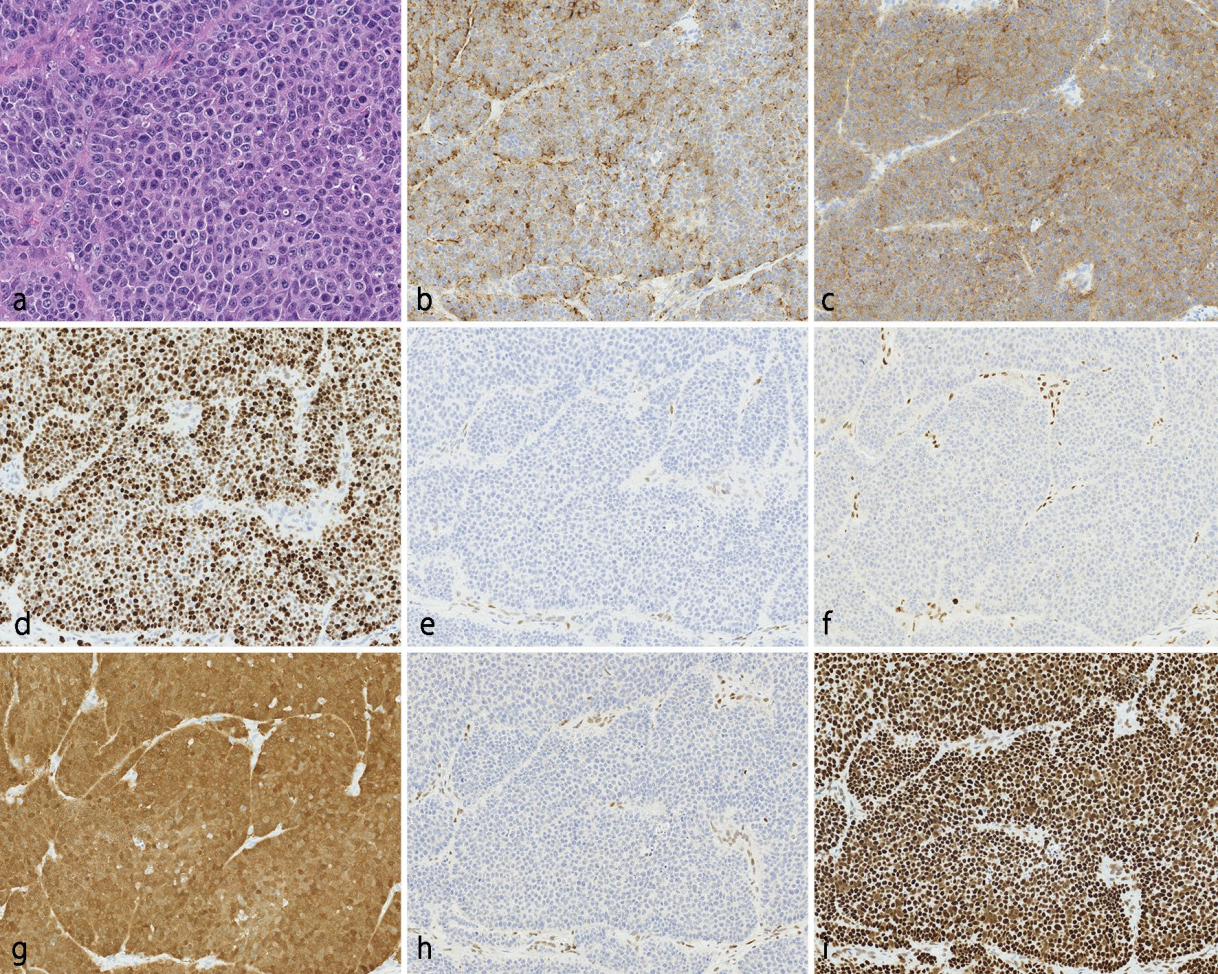
Histological and immunohistochemical findings for the sheet-like component. **a** The sheet-like component was composed of homogenous tumor cells characterized by scant cytoplasm and round nuclei with prominent nucleoli. The tumor showed intense staining for chromogranin A (**b**) and synaptophysin (**c**). **d** The Ki-67 labeling index was 72.8%. Tumor cells revealed complete deletion for p53 (**e**) and retinoblastoma (**f**), while non-tumor cells were sporadically positive. **g** Nuclear and cytoplasmic positive tumor cells for p16 were diffusely observed. **h** PMS2 expressions were completely deleted. **i** MLH6 was diffusely positive. Original magnification: **a** × 400; **b**–**i** × 200

**Fig. 4:**
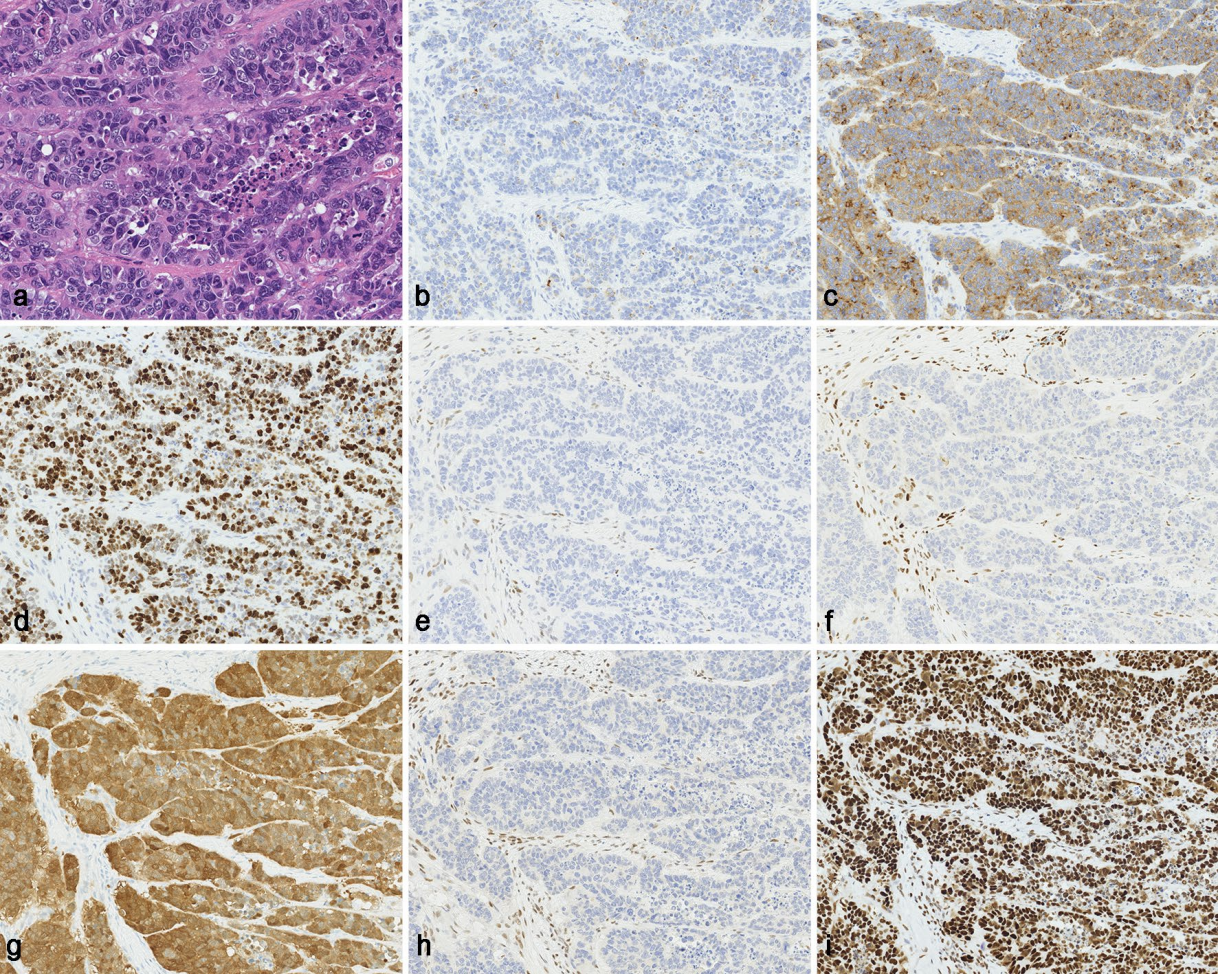
Histological and immunohistochemical findings for the pleomorphic component. **a** Tumor cells were diverse and showed pronounced nuclear pleomorphism. Necrosis was scattered in the tumor. **b** There were only a few chromogranin A positive cells. **c** Synaptophysin was diffusely positive. **d** The Ki-67 labeling index was 67.1%. The expressions of p53 **e** and retinoblastoma **f** protein were completely deleted in the tumor cells. **g** Intense staining for p16 was diffusely observed. **h** PMS2 showed complete deletion for the tumor. **i** MLH6 was diffusely positive. Original magnification: **a** × 400; **b**–**i** × 200

Immunohistochemically, the glandular component, as shown in Fig. 2, was positive for cytokeratin 7 (CK7) and MUC5AC, whereas it was negative for CK20, MUC2, MUC6, CDX2 and CD10. These findings supported the diagnosis of a gastric-type adenocarcinoma. The neuroendocrine markers for chromogranin A, synaptophysin, CD56 and insulinoma-associated protein 1 (INSM1) and the somatostatin receptor subtype (SSTR) 2 were all negative. The Ki-67 labeling index was 27.7%. An analysis looking for tumor suppressor gene proteins showed that p53 was not overexpressed or was completely deleted, p16 was negative, and both were normally expressed. By contrast, retinoblastoma (Rb) was abnormally expressed with complete deletion of the tumor cells. The sheet-like component, as shown in Fig. 3, was immunoreactive for chromogranin A, synaptophysin, and CD56, whereas INSM1 was negative. The Ki-67 labeling index was 72.8%. The pleomorphic component, as shown in Fig. 4, was also positive for synaptophysin and CD56, while chromogranin A was only marginally positive and INSM1 was negative. The Ki-67 labeling index was 67.1%. In these sheet-like and pleomorphic components, p53 and Rb exhibited aberrant expression showing completely deleted tumor cells. p16 also indicated aberrant expression showing diffusely positive tumor cells. Hormonal markers and SSTR2 were negative. These components were diagnosed as an NEC. In addition, MLH1 and PMS2 were completely deleted in the three morphological components, suggesting an MMR-deficient tumor. MSH2 and MSH6 expression was detected in all the components. The immunostaining results are shown in Table 2.

**Table 2:** Comparison of immunohistochemical findings in three morphological components

Markers	Glandular component	Sheet-like component	Pleomorphic component
Gastrointestinal differentiation markers			
Cytokeratin 7	+	–	–
Cytokeratin 20	–	–	–
MUC2	–	–	–
MUC5AC	+	–	–
MUC6	–	–	–
CDX2	–	–	–
CD10	–	–	–
Neuroendocrine differentiation markers			
Chromogranin A	–	+	+ (few)
Synaptophysin	–	+	+
CD56	–	+	+
INSM1	–	–	–
Somatostatin receptor			
SSTR2	–	–	–
Tumor suppressor gene proteins			
p53	+ (few)^a^	–, aberrant	–, aberrant
p16	–	+	+
Retinoblastoma	–, aberrant	–, aberrant	–, aberrant
Mismatch repair proteins			
MLH1	–, aberrant	–, aberrant	–, aberrant
PMS2	–, aberrant	–, aberrant	–, aberrant
MSH2	+	+	+
MSH6	+	+	+

+  positive, – negative, *INSM1* insulinoma-associated protein 1, *SSTR2* somatostatin receptor subtype 2

^a^This stainability represents the wild type status

A duodenal MANEC with MMR deficiency was diagnosed based on these morphological and immunohistochemical findings. Although we considered the possibility of the tumor originating from the accessory papilla, we judged it to have originated from the non-ampullary duodenum because it mainly spread to the wall of the duodenal bulb, and we observed no pancreaticobiliary system abnormalities in a clinical examination. The tumor was completely resected, although lymph node metastasis with a pleomorphic component was observed. The patient underwent adjuvant chemotherapy consisting of tegafur-gimeracil-oteracil potassium (TS-1) and has remained recurrence- and metastasis-free for two years.

## Discussion

We presented a rare case of non-ampullary duodenal MANEC composed of a conventional adenocarcinoma and an NEC. Of particular interest was that both the conventional adenocarcinoma and NEC showed MMR deficiency. We investigated the possibility of neuroendocrine differentiation and molecular abnormalities to characterize a tumor consisting of three morphologically distinct components. Several studies have shown that MMR deficiency was present in duodenal tumors from ampulla or non-ampulla duodenal tumors [21, 24–26]. However, large-scale reports of duodenal MMR-deficient tumors are scarce, and the exact frequency of this tumor, especially in the non-ampullary region, has not been revealed. There are also few reports of tumors showing neuroendocrine differentiation of the duodenum. Heetfeld et al. reported that of 167 gastroenteropancreatic NECs, 6 (4%) were primary duodenal NECs [27]. In addition, reports of intestinal MMR-deficient tumors with neuroendocrine differentiation are rare [5, 28, 29]. Sahnane et al. reported that out of 89 gastroenteropancreatic NECs and MANECs, 4 (4.5%) occurred in the duodenum, and one was an MMR-deficient tumor with the deletion of MSH2 and MSH6 immunohistochemically [29]. To the best our knowledge, this is the first report of a non-ampullary duodenal MANEC with MLH1 and PMS2 deletions.

The presence of three morphological components in this non-ampullary duodenal MANEC is an important clue to the tumorigenesis mechanism. The sheet-like component was morphologically similar to a well differentiated neuroendocrine neoplasm and was immunohistochemically diffusely positive for chromogranin A. This component, as well as the pleomorphic component, which was slightly positive for chromogranin A, also suggested genetic abnormalities in *TP53*, *Rb1*, and *p16* that are characteristic of NECs [9, 11]. We interpreted the sheet-like and pleomorphic components as regions with different degrees of neuroendocrine differentiation within the NEC. On the other hand, this MANEC had a gastric type glandular component. Non-ampullary duodenal adenocarcinomas with gastric differentiation have been reported to be more malignant and have a worse prognosis than those with intestinal differentiation [12–14]. The gastrointestinal differentiation of adenocarcinoma components in intestinal MANECs is not well documented, while it may be a necessary consideration in the duodenum, where tumors that differentiate into gastric types often occur. In addition, the glandular component showed no aberrant p53 expression, unlike the sheet-like and pleomorphic components. Several studies have addressed the idea that *TP53* mutation in gastric and colonic MANEC is shared by both adenocarcinomas and NECs [7, 8]. Taking these results into account, the MANEC we observed may be a collision tumor. On the other hand, all three components indicated MMR deficiency and aberrant Rb expression in this MANEC, suggesting that this tumor has a monoclonal origin. Minatsuki et al. reported that only 3 of 29 cases of early-stage non-ampullary duodenal adenocarcinoma showed aberrant p53 expression [13]. In non-ampullary duodenal MANECs, there may be cases in which *TP53* mutations are involved only in NEC tumorigenesis. Further studies with more cases are needed to elucidate the tumorigenesis mechanism of non-ampullary duodenal MANECs.

The present patient has remained recurrence- and metastasis-free for 2 years despite the presence of lymph node metastasis. MMR-deficient tumors have been shown to have a better prognosis than MMR-proficient tumors in small intestinal adenocarcinomas as well as colorectal adenocarcinomas [30, 31]. Although it was not a large series, La Rosa et al. reported that five patients with colorectal NECs or MANECs showing MMR deficiency were associated with a better prognosis than those with MMR proficiency [28]. In NECs or MANECs of the duodenum, it may be worthwhile to search for MMR-deficient tumors to evaluate the risk of recurrence.

We presented a rare case of a non-ampullary duodenal MANEC with MMR deficiency. We believe this to be a unique non-ampullary duodenal MANEC composed of a gastric-type adenocarcinoma and an NEC showing multistage differentiation. The course of MMR-deficient tumors in MANECs may be different from that of MMR-proficient tumors, and we propose that MMR-deficient tumors should be considered even in rare non-ampullary duodenal MANECs.

## References

[1] Klimstra DS, Klöppel G, La Rosa S, Rindi G (2019) Classification of neuroendocrine neoplasms of the digestive systems. In: WHO Classification of Tumours Editorial Board (eds) Digestive system tumours. International Agency for Research on Cancer, Lyon, pp16–19

[2] Milione M, Maisonneuve P, Pellegrinelli A, Grillo F, Albarello L, Spaggiari P, Vanoli A, Tagliabue G, Pisa E, Messerini L, Centonze G, Inzani F, Scarpa A, Papotti M, Volante M, Sessa F, Fazio N, Pruneri G, Rindi G, Solcia E, La Rosa S, Capella C (2018). Ki67 proliferative index of the neuroendocrine component drives MANEC prognosis. Endocr Relat Cancer.

[3] Milione M, Parente P, Grillo F, Zamboni G, Mastracci L, Capella C, Fassan M, Vanoli A (2021). Neuroendocrine neoplasms of the duodenum, ampullary region, jejunum and ileum. Pathologica.

[4] Konukiewitz B, Kasajima A, Schmitt M, Schwamborn K, Groll T, Schicktanz F, Delbridge C, Schütze LM, Wilhelm D, Lang C, Lange S, Foersch S, Jank P, Steiger K, Werder AV, Denkert C, Weichert W, Klöppel G, Jesinghaus M (2021). Neuroendocrine differentiation in conventional colorectal adenocarcinomas: incidental finding or prognostic biomarker?. Cancers (Basel).

[5] Jesinghaus M, Schmitt M, Lang C, Reiser M, Scheiter A, Konukiewitz B, Steiger K, Silva M, Tschurtschenthaler M, Lange S, Foersch S, Becker KF, Saur D, Friess H, Halfter K, Engel J, Boxberg M, Pfarr N, Wilhelm D, Weichert W (2021). Morphology matters: a critical reappraisal of the clinical relevance of morphologic criteria from the 2019 WHO classification in a large colorectal cancer cohort comprising 1004 cases. Am J Surg Pathol.

[6] Kim KM, Kim MJ, Cho BK, Choi SW, Rhyu MG (2002). Genetic evidence for the multi-step progression of mixed glandular-neuroendocrine gastric carcinomas. Virchows Arch.

[7] Scardoni M, Vittoria E, Volante M, Rusev B, Bersani S, Mafficini A, Gottardi M, Giandomenico V, Malleo G, Butturini G, Cingarlini S, Fassan M, Scarpa A (2014). Mixed adenoneuroendocrine carcinomas of the gastrointestinal tract: targeted next-generation sequencing suggests a monoclonal origin of the two components. Neuroendocrinology.

[8] Koh J, Nam SK, Kwak Y, Kim G, Kim KK, Lee BC, Ahn SH, Park DJ, Kim HH, Park KU, Kim WH, Lee HS (2021). Comprehensive genetic features of gastric mixed adenoneuroendocrine carcinomas and pure neuroendocrine carcinomas. J Pathol.

[9] Konukiewitz B, Schlitter AM, Jesinghaus M, Pfister D, Steiger K, Segler A, Agaimy A, Sipos B, Zamboni G, Weichert W, Esposito I, Pfarr N, Klöppel G (2017). Somatostatin receptor expression related to TP53 and RB1 alterations in pancreatic and extrapancreatic neuroendocrine neoplasms with a Ki67-index above 20. Mod Pathol.

[10] Hijioka S, Hosoda W, Matsuo K, Ueno M, Furukawa M, Yoshitomi H, Kobayashi N, Ikeda M, Ito T, Nakamori S, Ishii H, Kodama Y, Morizane C, Okusaka T, Yanagimoto H, Notohara K, Taguchi H, Kitano M, Yane K, Maguchi H, Tsuchiya Y, Komoto I, Tanaka H, Tsuji A, Hashigo S, Kawaguchi Y, Mine T, Kanno A, Murohisa G, Miyabe K, Takagi T, Matayoshi N, Yoshida T, Hara K, Imamura M, Furuse J, Yatabe Y, Mizuno N (2017). Rb loss and KRAS mutation are predictors of the response to platinum-based chemotherapy in pancreatic neuroendocrine neoplasm with grade 3: a Japanese multicenter pancreatic NEN-G3 study. Clin Cancer Res.

[11] Yachida S, Vakiani E, White CM, Zhong Y, Saunders T, Morgan R, de Wilde RF, Maitra A, Hicks J, Demarzo AM, Shi C, Sharma R, Laheru D, Edil BH, Wolfgang CL, Schulick RD, Hruban RH, Tang LH, Klimstra DS, Iacobuzio-Donahue CA (2012). Small cell and large cell neuroendocrine carcinomas of the pancreas are genetically similar and distinct from well-differentiated pancreatic neuroendocrine tumors. Am J Surg Pathol.

[12] Ushiku T, Arnason T, Fukayama M, Lauwers GY (2014). Extra-ampullary duodenal adenocarcinoma. Am J Surg Pathol.

[13] Minatsuki C, Yamamichi N, Inada KI, Takahashi Y, Sakurai K, Shimamoto T, Tsuji Y, Shiogama K, Kodashima S, Sakaguchi Y, Niimi K, Ono S, Niwa T, Ohata K, Matsuhashi N, Ichinose M, Fujishiro M, Tsutsumi Y, Koike K (2018). Expression of gastric markers is associated with malignant potential of nonampullary duodenal adenocarcinoma. Dig Dis Sci.

[14] Yoshida M, Shimoda T, Abe M, Kakushima N, Kawata N, Takizawa K, Ono H, Sugino T (2019). Clinicopathological characteristics of non-ampullary duodenal tumors and their phenotypic classification. Pathol Int.

[15] Le DT, Uram JN, Wang H, Bartlett BR, Kemberling H, Eyring AD, Skora AD, Luber BS, Azad NS, Laheru D, Biedrzycki B, Donehower RC, Zaheer A, Fisher GA, Crocenzi TS, Lee JJ, Duffy SM, Goldberg RM, de la Chapelle A, Koshiji M, Bhaijee F, Huebner T, Hruban RH, Wood LD, Cuka N, Pardoll DM, Papadopoulos N, Kinzler KW, Zhou S, Cornish TC, Taube JM, Anders RA, Eshleman JR, Vogelstein B, Diaz LA (2015). PD-1 blockade in tumors with mismatch-repair deficiency. N Engl J Med.

[16] Hampel H, Frankel WL, Martin E, Arnold M, Khanduja K, Kuebler P, Nakagawa H, Sotamaa K, Prior TW, Westman J, Panescu J, Fix D, Lockman J, Comeras I, de la Chapelle A (2005). Screening for the Lynch syndrome (hereditary nonpolyposis colorectal cancer). N Engl J Med.

[17] Shia J (2008). Immunohistochemistry versus microsatellite instability testing for screening colorectal cancer patients at risk for hereditary nonpolyposis colorectal cancer syndrome. Part I. The utility of immunohistochemistry. J Mol Diagn.

[18] Shia J, Stadler Z, Weiser MR, Rentz M, Gonen M, Tang LH, Vakiani E, Katabi N, Xiong X, Markowitz AJ, Shike M, Guillem J, Klimstra DS (2011). Immunohistochemical staining for DNA mismatch repair proteins in intestinal tract carcinoma: how reliable are biopsy samples?. Am J Surg Pathol.

[19] Le DT, Durham JN, Smith KN, Wang H, Bartlett BR, Aulakh LK, Lu S, Kemberling H, Wilt C, Luber BS, Wong F, Azad NS, Rucki AA, Laheru D, Donehower R, Zaheer A, Fisher GA, Crocenzi TS, Lee JJ, Greten TF, Duffy AG, Ciombor KK, Eyring AD, Lam BH, Joe A, Kang SP, Holdhoff M, Danilova L, Cope L, Meyer C, Zhou S, Goldberg RM, Armstrong DK, Bever KM, Fader AN, Taube J, Housseau F, Spetzler D, Xiao N, Pardoll DM, Papadopoulos N, Kinzler KW, Eshleman JR, Vogelstein B, Anders RA, Diaz LA (2017). Mismatch repair deficiency predicts response of solid tumors to PD-1 blockade. Science.

[20] Latham A, Srinivasan P, Kemel Y, Shia J, Bandlamudi C, Mandelker D, Middha S, Hechtman J, Zehir A, Dubard-Gault M, Tran C, Stewart C, Sheehan M, Penson A, DeLair D, Yaeger R, Vijai J, Mukherjee S, Galle J, Dickson MA, Janjigian Y, O'Reilly EM, Segal N, Saltz LB, Reidy-Lagunes D, Varghese AM, Bajorin D, Carlo MI, Cadoo K, Walsh MF, Weiser M, Aguilar JG, Klimstra DS, Diaz LA, Baselga J, Zhang L, Ladanyi M, Hyman DM, Solit DB, Robson ME, Taylor BS, Offit K, Berger MF, Stadler ZK (2019). Microsatellite instability is associated with the presence of Lynch syndrome pan-cancer. J Clin Oncol.

[21] Planck M, Ericson K, Piotrowska Z, Halvarsson B, Rambech E, Nilbert M (2003). Microsatellite instability and expression of MLH1 and MSH2 in carcinomas of the small intestine. Cancer.

[22] González I, Goyal B, Xia MD, Pai RK, Ma C (2019). DNA mismatch repair deficiency but not ARID1A loss is associated with prognosis in small intestinal adenocarcinoma. Hum Pathol.

[23] Ladabaum U, Ford JM, Martel M, Barkun AN (2015). American gastroenterological association technical review on the diagnosis and management of Lynch syndrome. Gastroenterology.

[24] Sessa F, Furlan D, Zampatti C, Carnevali I, Franzi F, Capella C (2007). Prognostic factors for ampullary adenocarcinomas: tumor stage, tumor histology, tumor location, immunohistochemistry and microsatellite instability. Virchows Arch.

[25] Fu T, Pappou EP, Guzzetta AA, Jeschke J, Kwak R, Dave P, Hooker CM, Morgan R, Baylin SB, Iacobuzio-Donahue CA, Wolfgang CL, Ahuja N (2012). CpG island methylator phenotype-positive tumors in the absence of MLH1 methylation constitute a distinct subset of duodenal adenocarcinomas and are associated with poor prognosis. Clin Cancer Res.

[26] Watari J, Mitani S, Ito C, Tozawa K, Tomita T, Oshima T, Fukui H, Kadowaki S, Natsume S, Senda Y, Tajika M, Hara K, Yatabe Y, Shimizu Y, Muro K, Morimoto T, Hirota S, Das KM, Miwa H (2019). Molecular alterations and PD-L1 expression in non-ampullary duodenal adenocarcinoma: associations among clinicopathological, immunophenotypic and molecular features. Sci Rep.

[27] Heetfeld M, Chougnet CN, Olsen IH, Rinke A, Borbath I, Crespo G, Barriuso J, Pavel M, O'Toole D, Walter T, other Knowledge Network members (2015). Characteristics and treatment of patients with G3 gastroenteropancreatic neuroendocrine neoplasms. Endocr Relat Cancer.

[28] La Rosa S, Marando A, Furlan D, Sahnane N, Capella C (2012). Colorectal poorly differentiated neuroendocrine carcinomas and mixed adenoneuroendocrine carcinomas: insights into the diagnostic immunophenotype, assessment of methylation profile, and search for prognostic markers. Am J Surg Pathol.

[29] Sahnane N, Furlan D, Monti M, Romualdi C, Vanoli A, Vicari E, Solcia E, Capella C, Sessa F, La Rosa S (2015). Microsatellite unstable gastrointestinal neuroendocrine carcinomas: a new clinicopathologic entity. Endocr Relat Cancer.

[30] Latham A, Shia J, Patel Z, Reidy-Lagunes DL, Segal NH, Yaeger R, Ganesh K, Connell L, Kemeny NE, Kelsen DP, Hechtman JF, Nash GM, Paty PB, Zehir A, Tkachuk KA, Sheikh R, Markowitz AJ, Mandelker D, Offit K, Berger MF, Cercek A, Garcia-Aguilar J, Saltz LB, Weiser MR, Stadler ZK (2021). Characterization and clinical outcomes of DNA mismatch repair-deficient small bowel adenocarcinoma. Clin Cancer Res.

[31] Aparicio T, Svrcek M, Henriques J, Afchain P, Lièvre A, Tougeron D, Gagniere J, Terrebonne E, Piessen G, Legoux JL, Lecaille C, Pocard M, Gornet JM, Zaanan A, Lavau-Denes S, Lecomte T, Deutsch D, Vernerey D, Puig PL (2021). Panel gene profiling of small bowel adenocarcinoma: results from the NADEGE prospective cohort. Int J Cancer.

